# New World Health Organization reference values for semen analysis: where do we stand?

**DOI:** 10.1590/S1679-45082013000200023

**Published:** 2013

**Authors:** Marcelo Vieira

**Affiliations:** * Projeto ALFA – São Paulo, SP, Brazil

**Keywords:** Semen Analysis, Infertility, Reference values

## Abstract

Semen analysis is of paramount importance to study potential male fertility, couple's infertility, the effects of gonadotoxic agents on spermatogenesis and as follow-up test during treatment of male infertility. Since 1987, the World Health Organization proposes the standardization of this test and its reference values based on population-based data. The latest version of the World Health Organization guidelines was published in 2010. It introduced a new methodology that produced new references values, which triggered a discussion that lies inconclusive. We revised the original World Health Organization paper focusing on methodological changes and its results, the new references values and their impact on clinical practice.

## RESUMO

A análise seminal é o exame fundamental para a caracterização do potencial de fertilidade masculina, na investigação da infertilidade conjugal, para o acompanhamento do tratamento das causas de infertilidade masculina e para o estudo dos efeitos de gonadotóxicos sobre a espermatogênese. Desde 1987, a Organização Mundial da Saúde propõe a padronização da execução do exame e de seus valores de normalidade, baseados em avaliações populacionais. Em 2010, ela publicou seu último manual, contendo mudanças metodológicas que determinaram novos valores de normalidade e abrindo uma discussão que persiste na literatura. Neste artigo, revisaremos o trabalho original e os principais aspectos a respeito da mudança na metodologia, os novos valores e a repercussão na prática clínica.

Semen analysis is the initial laboratory test conducted to examine the role played by men in couple's infertility. Since 1987, the World Health Organization (WHO) publishes guidelines standardizing procedures for assessing human semen and proposing reference values according to data from men around the world^(1)^. However, it is very difficult to establish values that express fertility because many factors can affect the test result – lack of standardization or proper laboratory training, regional and socioeconomic differences between countries and continents that affect fertility, and finally the fact that fertility depends on two people and the role played by women may be overlooked^(2)^.

Until 1999, WHO guidelines were based on data that came from several laboratories that used different methodologies and examined different male populations, not supported by standardized methods or without the definition of fertile population. This could produce misidentification of normal values, and normal men could be considered infertile if reference values were too high and did not reflect the reality or if they were below what is necessary to promote pregnancy. The male population studied until 2010 included men without proven paternity, patients of human reproduction clinics that sought treatment, semen donors and vasectomy candidates. Semen donors can be fertile and vasectomy candidates are very likely to be fertile, although there is no data about how long it took for their partners to get pregnant^(1)^.

The new references values, published in the 2010 WHO guidelines, are lower than those published in 1999. There were questions raised about the reduced fertility that is affecting the whole world, biased changes in the data collected, failure of the study methodology, and determination of reference values^(2–4)^.

The virtue and the main criticism to this study lies on the method used for data collection, selection of groups studied, and the determination of normal reference values^(3,4)^.

The objective of this paper is to highlight the strengths and weaknesses of this methodology in determining the new reference values to physicians engaged in treating male partners of infertile couples.

Reference values can be given by samples from single individuals or by samples from a group of healthy individuals (or individuals without the condition). The range of normal values is determined by fifth centiles, with its lower and upper limits determined by percentiles 2.5 and 95. Identification of healthy individuals, i.e., fertile individuals, was based on the definition of infertility (unable to conceive after having 12 months of unprotected intercourse). Data came from 1,953 samples, 5 studies, 8 countries on 3 continents with timeto-pregnancy up to and including 12 months^(1)^. Data from three other groups were used for comparison: (1) “unscreened” men from the general population or young volunteers participating in hormonal contraception studies, considered representatives of the general population (965 samples, 7 studies, 5 countries, 3 continents); (2) “screened” men from different origins, of unknown fertility but with semen analysis within reference values (934 samples, 4 studies, 4 countries, 3 continents, 2 WHO multinational studies); and (3) fertile men with unknown TTP, representing the group and all ranges of fecundity – normal, moderately or severely impaired (817 samples, 2 studies, 2 continents, 2 WHO multinational studies). This methodology succeeds in having four groups with different potential fertility rates, and most importantly, a normal group of men who have fathered a child within one year. Lower reference values were given by data from men whose partners had TTP ≤ 12 months: semen volume, 1.5mL (1.4 – 1.7); total sperm number, 39 million per ejaculate (33-46); sperm concentration, 15 million per mL (12 – 16); vitality, 58% live (55 – 63); progressive motility, 32% (31 – 34); total (progressive and non-progressive) motility, 40% (38 – 42); morphologically normal forms, 4% (3 – 4). Semen quality analysis demonstrated that the semen of men whose partners had TTP ≤ 12 months was superior to that of men from the general population and normozoospermic men^(1)^.

Despite the advanced methodology used in the latest guidelines, there is still a debate about the use of data obtained from studies that used different methodologies for semen analysis, particularly regarding sperm morphology. Another point of debate is the representativeness of the sample population- most data come from the Northern hemisphere, without including South America, Africa and Asia. The sample is strongly based on data from men from Australia, Northern Europe and North America whose partners had TTP ≤ 12 months. The fact that the sample is based on a single sample of semen is also strongly criticized considering major temporal variability of the quality of the semen from the same man^(3)^.

The new reference values have clinical impact because they now classify men who were previously considered infertile as normal. This could delay the indication of fertility treatment, impairing its outcome^(3,4)^. From the practical standpoint, the indication of varicocele surgery, for example, has changed according to changes of reference values and this has had impact on legal issues and treatment payment by health insurance companies. The new reference values proposed should be examined together with couple's assessment, and not alone. We expect clinical laboratories to check the quality of tests, implement standardized procedures and follow these new reference values in order to provide answers to our current questions.
